# Critical distance of the sacroiliac joint for open reduction using screw fixation for traumatic sacroiliac joint diastasis: a retrospective study

**DOI:** 10.1186/s13018-024-04759-z

**Published:** 2024-04-27

**Authors:** Ruei-Shyuan Chien, I-Jung Chen, Chih-Yang Lai, Jui‑Ping Chen, Yi-Hsun Yu

**Affiliations:** Division of Orthopedic Traumatology, Department of Orthopedic Surgery, Musculoskeletal Research Center, Chang Gung Memorial Hospital, Linkou Branch, Chang Gung University, 5, Fu-Hsin St. Kweishan, Taoyüan, 33302 Taiwan

**Keywords:** Pelvic fracture, Sacroiliac joint, Iliosacral screw, Open reduction, Radiological outcome

## Abstract

**Background:**

Osteosynthesis for sacroiliac joint (SIJ) diastasis using an iliosacral screw (ISS) and a trans-iliac-trans-sacral screw (TITSS) can be performed using a closed or an open method. However, no clear indication for open reduction has been established.

**Methods:**

Data on patients with unilateral traumatic SIJ diastasis who underwent ISS and TITSS fixation were retrospectively collected and separated into groups according to the reduction method: closed reduction group (C group) and open reduction group (O group). Demographic data and perioperative image assessments were compared between the groups. The critical distance of the SIJ was identified to elucidate the indication for open reduction of the diastatic SIJ.

**Results:**

Fifty-six patients met the inclusion criteria over a 3-year period. There was no significant difference in the reduction quality of pelvic ring injuries between the groups, according to Matta’s and Lefaivre’s criteria. The improvement in the SIJ distance was significantly greater in the O group than in the C group in the axial plane on multiplanar computed tomography (*p* = 0.021). This model predicted that a difference of > 3.71 mm between the injured and healthy SIJ was a recommended indication for open reduction, with an area under the curve of 0.791 (95% confidence interval 0.627–0.955, *p* = 0.004).

**Conclusions:**

Open reduction for SIJ diastasis might achieve better reduction quality than does closed reduction in the axial plane in selected cases. When the difference between the injured and healthy SIJ was wider than 3.71 mm, open reduction was recommended for satisfactory radiological outcomes.

## Background

The integrity of the posterior weight-bearing sacroiliac joint (SIJ) contributes to pelvic ring stability [1]. SIJ diastasis, a crucial pattern in posterior pelvic ring injuries, often results from high-energy trauma, and the soft tissue surrounding the pelvis is often severely contused and disrupted [2, 3]. Pelvic malunion can cause severe sequelae if untreated, which include leg length discrepancy, posterior pelvic pain, sitting imbalance, and back pain [4, 5].

The optimal treatment for the complex SIJ diastasis remains a challenge for orthopedic surgeons [6]. Minimizing the gap between the ilium and sacrum to the anatomical positions is one of the key goals for achieving satisfactory radiological and functional outcomes [7–9]. Therefore, the aim of operative treatment is to restore normal anatomy by rigid fixation to enable early mobilization in order to obtain functional recovery.

Different fixation methods have been developed for SIJ diastasis [10–12]. Osteosynthesis is achieved by an open or a closed method followed by internal fixation, including iliosacral screw (ISS) fixation, trans-iliac–trans-sacral screw (TITSS) fixation, sacral bar fixation, spinopelvic osteosynthesis, posterior tension band plating, and anterior SIJ plating [10, 13–16]. Minimally invasive procedures using percutaneous screws, such as ISS or TITSS fixation, are preferred for osteosynthesis in cases of SIJ diastasis, contributing to a decrease in operating time, surgical bleeding, and soft tissue disruption and preventing nonunion [17, 18].

Reduction of the dislocated SIJ using an open or a closed method should be performed prior to percutaneous screw fixation. Compared with the open method, closed reduction is superior because it reduces intraoperative blood loss and lowers the infection rate without compromising the reduction quality [19–21]. However, in some injury patterns such as crescent fracture and completely dislocated SIJ, closed reduction is anticipated to fail and an open approach is recommended to achieve anatomical reduction [22]. Despite this, an absolute indication for open reduction of the SIJ is lacking.

Since there is no quantitative evidence that open reduction should be advocated for widening of the SIJ, the actual indication for open reduction for the diastatic SIJ should be explored. The primary aim of our study was to compare radiological outcomes after osteosynthesis for SIJ diastasis using closed or open reduction, followed by ISS and TITSS fixation. Additionally, the critical distance of the widened SIJ that supports open reduction was elucidated.

## Methods

### Patient allocation

Medical records of patients who were diagnosed with pelvic ring injury (PRI) and underwent osteosynthesis between January 2017 and December 2021 were retrospectively collected from the fracture registration database of our institute. The inclusion criteria were as follows: (1) unilateral SIJ diastasis and percutaneous screw(s) fixation (ISS, TITSS, or both) through a closed or an open reduction technique; (2) management for SIJ diastasis as the primary or first reduction for PRI (before reduction and fixation of anterior pelvic ring injury); and (3) completeness of medical and radiological records for at least 12 months. Patients under 18 years of age and those who underwent screw fixation as an emergent resuscitative procedure (an antishock screw) or screw fixation with bone cement augmentation (for fragility fracture or pathological fracture of the pelvic ring) were excluded. Medical records and preoperative and postoperative radiological images were meticulously reviewed and analyzed. All operative procedures were performed by a single senior surgeon.

### Perioperative protocol and surgical technique

**Fig. 1:**
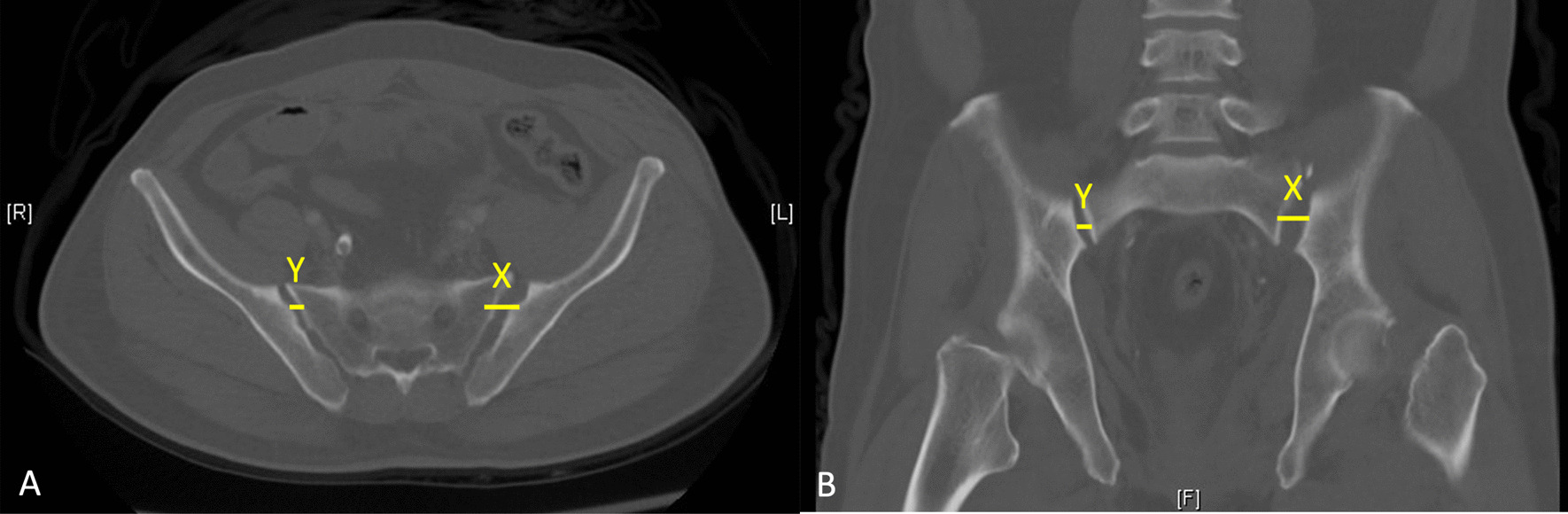
Maximal SIJ diastasis (X) assessment by preoperative mpCT in **A** axial and **B** coronal views. Distance Y is defined as healthy SIJ distance. X–Y = difference between healthy and injured SIJ distance. *SIJ* Sacroiliac joint, *mpCT* Multiplanar computed tomography

The patients were admitted to the ordinary ward or intensive care unit as required after primary resuscitation. Osteosynthesis for PRI was performed as soon as possible after the patient was hemodynamically stabilized. Prior to definite osteosynthesis, standard radiological images including pelvic radiography (anteroposterior [AP], inlet, and outlet views) and multiplanar computed tomography (mpCT) were obtained for surgical planning. If a pelvic binder was applied during initial image evaluations, the X-rays and mpCT were systematically repeated before surgery to avoid potential underclassification. We collected information on maximal SIJ diastasis along with the difference between healthy and injured SIJ from mpCT in both axial and coronal views (Fig. 1). Patients who were not indicated for ISS or TITSS because of conditions such as sacral dysmorphisms and a shallow bony corridor were excluded for the planning screw placement.

The operation was performed under general anesthesia with the patients in the supine or prone position, depending on the planned procedures and concomitant injuries observed, on a radiolucent table (Modular Table System^©^; Mizuho OSI, California, USA). The supine position was preferred for those showing contraindications for the prone position. Before beginning sterile draping, the patients’ injuries were evaluated under anesthesia to determine the necessity to upgrade the fracture pattern and change the surgical plan. Real-time fluoroscopic images including AP, inlet, outlet, Judet oblique views, and sacrum lateral views were examined prior to surgical draping to ensure that all images could be obtained clearly without limitations. The intraoperative images were obtained by using a single-arm fluoroscopic intensifier (Ziehm Solo; Ziehm Imaging GmbH, Nuremberg, Germany).

**Fig. 2:**
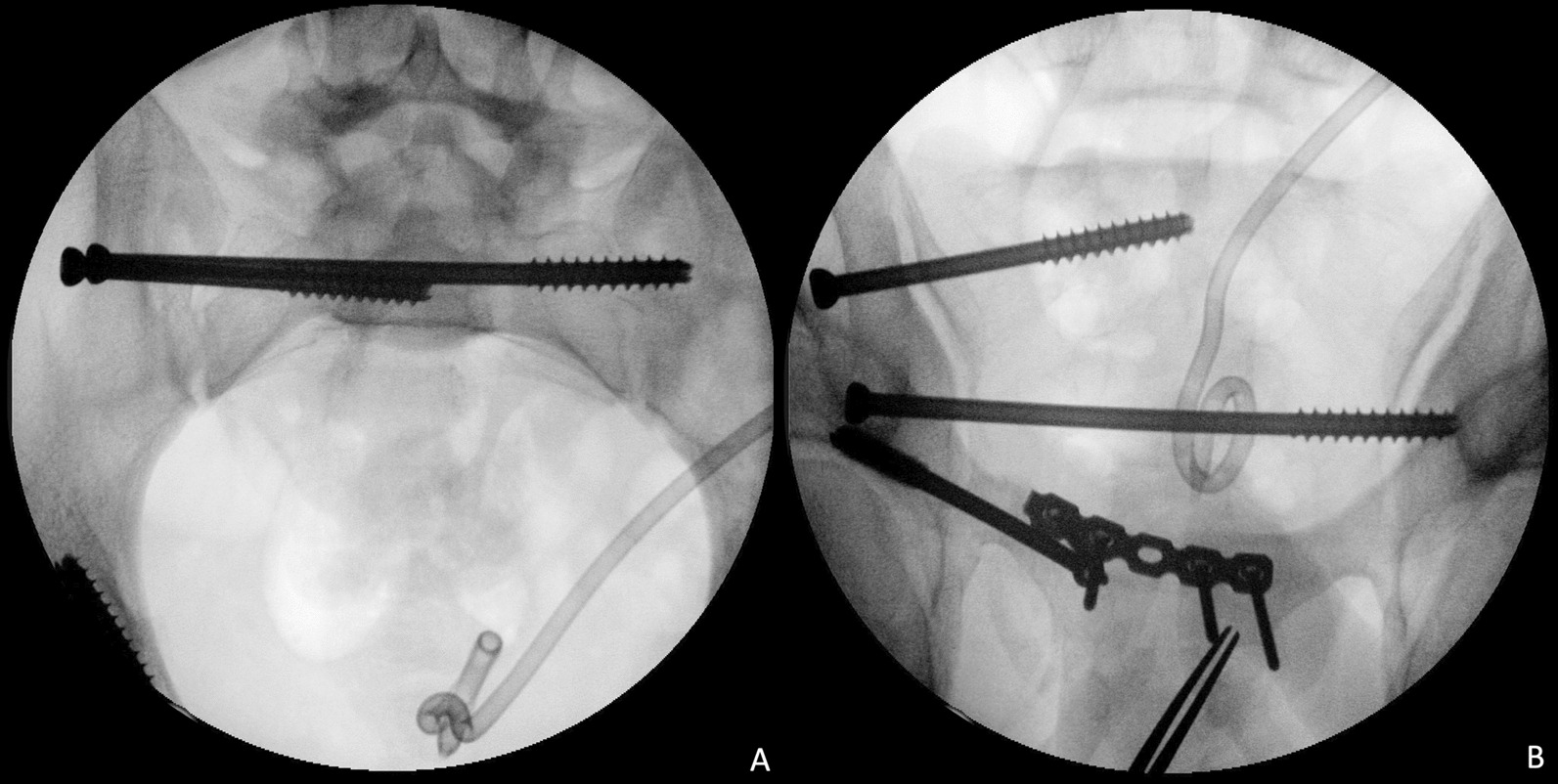
Fluoroscopy images for closed reduction by direct compression of SIJ and fixation with ISS/TITS. **A** Inlet view. **B** Outlet view. *ISS* Iliosacral screw, *TITSS* Trans-iliac-trans-sacral screw

The osteosynthesis strategy for anterior and posterior PRI can be performed simultaneously (supine position) or sequentially (first prone and then supine positions). However, posterior pelvic ring reduction and fixation were performed before the anterior pelvic ring procedure. For the closed reduction technique, the SIJ was reduced by the direct compression force provided by an appropriately placed ISS perpendicular to the orientation of the SIJ (Fig. 2). The TITSS was implanted after ISS fixation for the purpose of augmented fixation.

**Fig. 3:**
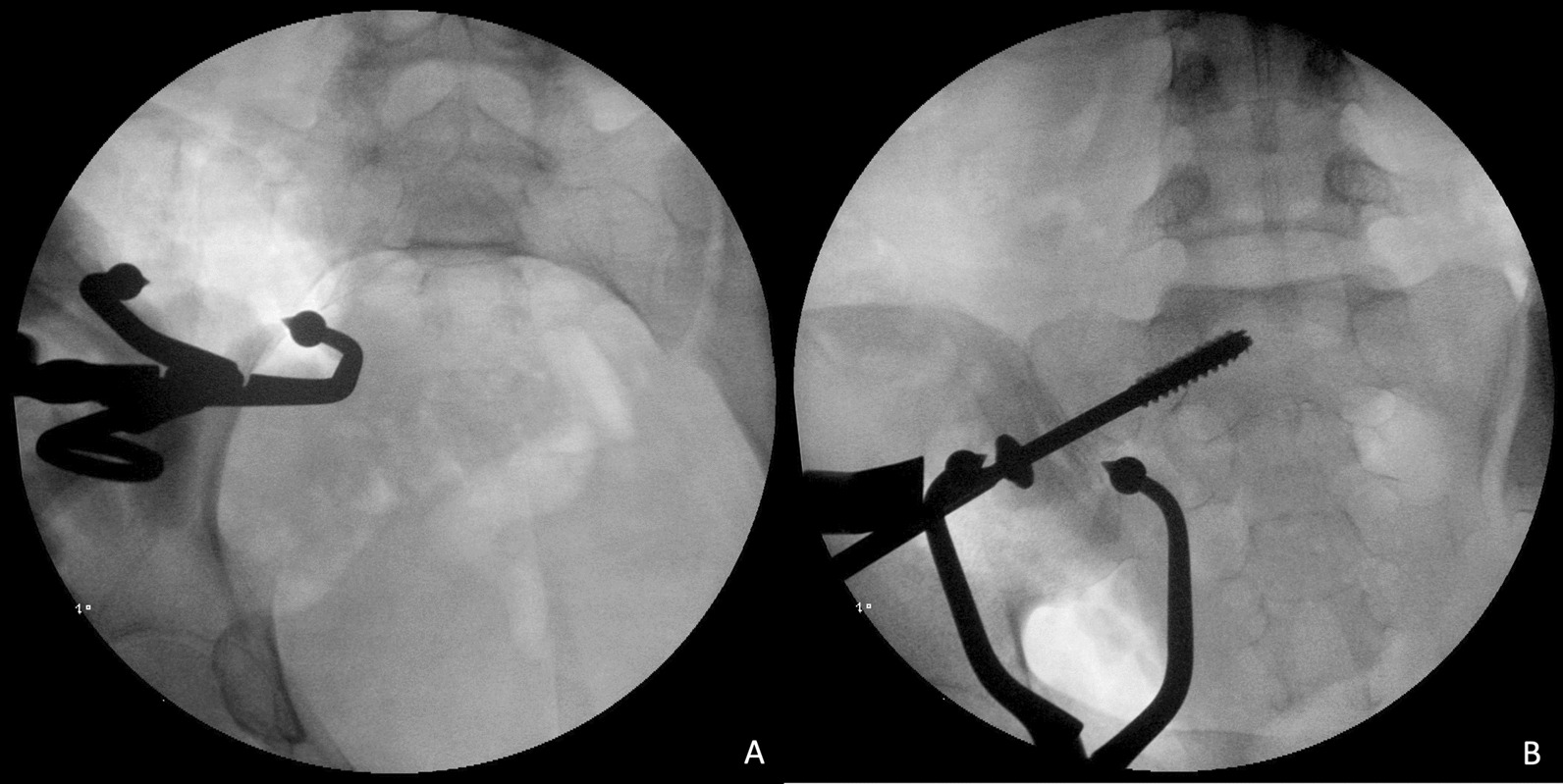
Fluoroscopy images for open reduction and fixation with ISS. **A** Inlet view. **B** Outlet view. *ISS* Iliosacral screw

On the other hand, the surgical approach could be either from the anterior (through the lateral window of the ilioinguinal approach in the supine position) or dorsal (a 5 cm longitudinal incision direct or 1 cm lateral to the posterior superior iliac spine in the prone position) side for the open reduction technique. Using the dorsal approach, the angled Matta clamp (DePuy Synthes, Paoli, PA, USA) was applied with one jaw on the hooked on the anterior cortex of the sacrum and the other on the dorsal cortex of the ilium (Fig. 3). After reduction of the SIJ was confirmed via fluoroscopic images, a provisional Kirschner wire was inserted percutaneously under real-time fluoroscopic examination. A 7.0-mm cannulated screw (Syntec Technology Co., Hsinchu, Taiwan) was applied as the target implant of the ISS. Whenever an additional screw was necessary to provide stability to the posterior pelvic ring, a cannulated screw (TITSS 7.0 mm; Syntec Technology Co., Hsinchu, Taiwan) with an orientation parallel to the groundline wound was applied. The radiation dose and time were recorded during percutaneous screw osteosynthesis.

Following completion of reduction and fixation of the SIJ, anterior pelvic ring reduction and fixation were performed. Intramedullary screw (Syntec Technology Co., Hsinchu, Taiwan) fixation was planned for the pubic rami fractures, for which closed reduction was performed in the prone (antegrade fashion) or supine (antegrade or retrograde fashion) position. When the anterior pelvic injury was indicated for open reduction (pubic symphysis diastasis or failed/inadequate reduction of the pubic rami fractures), a Pfannenstiel incision was placed and plate osteosynthesis (3.5-mm reconstruction plate, DePuy Synthes, Paoli, PA, USA) was performed.

### Image assessment protocol

Identical postoperative imaging examinations were performed to examine the reduction quality of the pelvic ring and position of the implants using radiographs, including AP, inlet, and outlet views, and mpCT. Radiological interpretation was performed by an independent medical doctor, who was not involved in the surgeries, using the Picture Archiving and Communication System (Centricity Enterprise Web V3.0; GE Healthcare, Chicago, USA), and the interpretations were re-examined repeatedly by two other senior orthopedic surgeons.

**Fig. 4:**
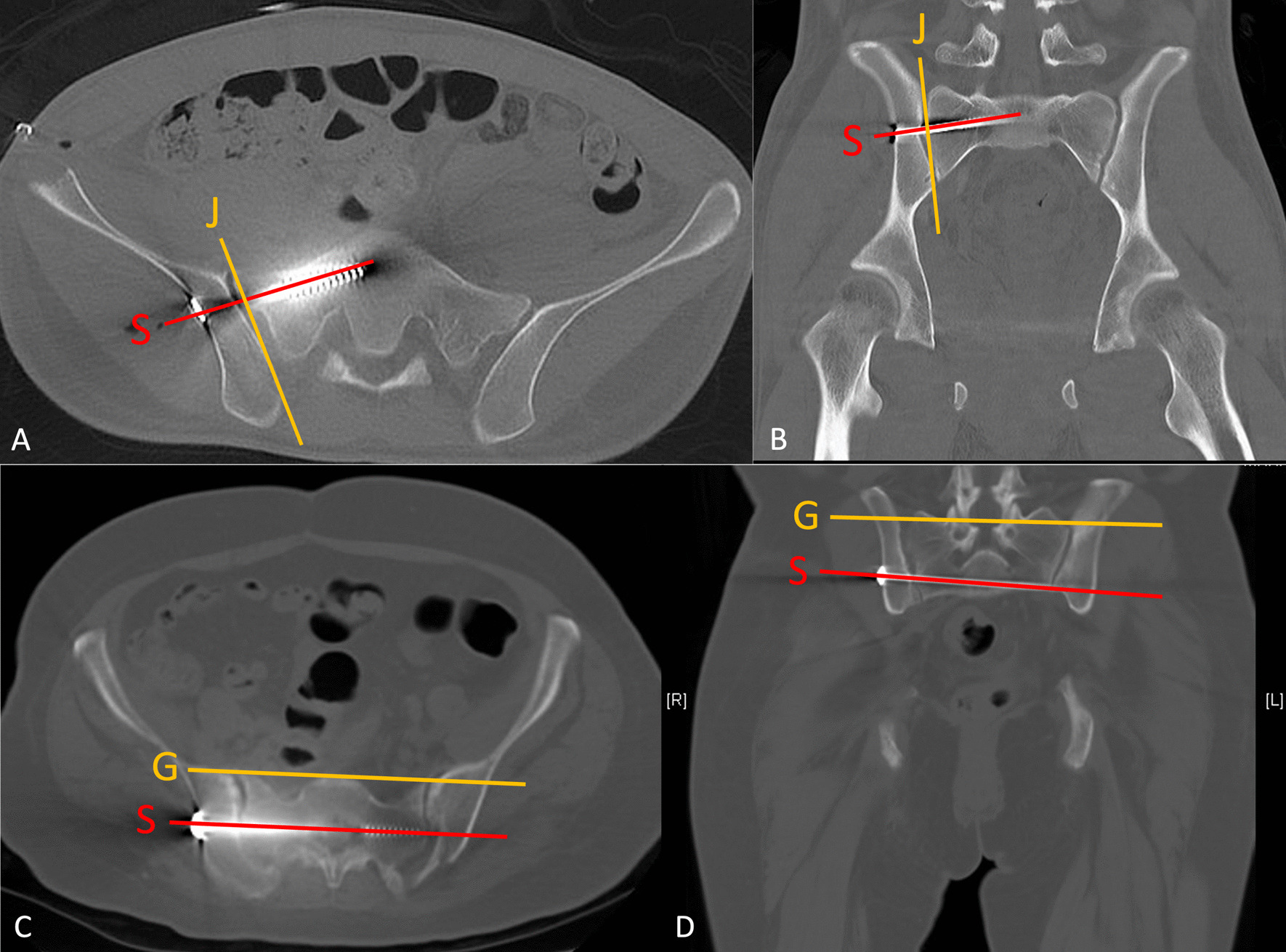
Ideal angles between ISS (line S) and the sacroiliac joint (line J). The ideal angles are 90° in both **A** axial and **B** coronal views. The actual angles that deviated from these ideal orientations are defined as the ADIO of ISS. The ideal angle between TITSS (line S) and groundline (line G) is 0° in both **C** axial and **D** coronal views. The actual angles that deviated from these ideal orientations are defined as the ADIO of TITS. The ISS, TITSS, and ADIO deviated from the ideal orientation. *ISS* Iliosacral screw, *TITSS* Trans-iliac-trans-sacral screw, *ADIO* Angles deviated from the ideal orientation

Several classifications and grading systems for fracture patterns, reduction quality, and screw positions were used in this study. The PRI was classified using the Arbeitsgemeinschaft für Osteosynthesefragen (AO) classification system [23]. The reduction qualities after osteosynthesis were evaluated from radiographs, including axial and coronal views from mpCT. We adapted the criteria of Matta/Tornetta and Lefaivre to interpret the reduction quality [24–26]. Accordingly, the reduction quality of the pelvic ring injury was classified as excellent, good, fair, or poor. For the ISS, we examined the angles between the screw and the SIJ obtained in the axial and coronal views of the mpCT. The angular differences between the implanted screw and the ideal orientation of the ISS, which should be perpendicular to the SIJ in each view, were measured and identified as the angles deviated from the ideal orientation (ADIO) (Fig. 4) [27]. For TITSS, the angular difference between the implanted screw and the ideal orientation, which should be parallel to the groundline in both axial and coronal views of the mpCT scan, were recorded.

### Statistical analysis

Because of the retrospective nature of the study, Pearson’s correlation analysis was first employed to identify potential variables that were correlated to outcome measurements in terms of a reduction in the SIJ distance. The chi-squared test was used to analyze categorical data. The t-test was used for between-group comparisons of numerical data. Statistical significance was set as *p* < 0.05. The cutoff point value was defined as the maximum Youden’s index. Statistical analyses were performed using SPSS 26.0 program for Windows (IBM SPSS Statistics for Windows, Version 26.0; IBM Corp, NY, USA).

## Results

Fifty-six patients with unilateral SIJ injuries underwent percutaneous screw fixation between January 2017 and December 2021. Table 1 shows that the presence of sacral fracture and the SIJ reduction method were significantly correlated with a reduced SIJ distance on axial and coronal views of mpCT (*p* = 0.031 and 0.001 for sacral fracture and *p* = 0.008 and 0.025 for SIJ reduction method, respectively). This result was consistent with our hypothesis that the indication for closed/open reduction may be correlated with the reduction in the SIJ distance.

**Table 1:** Pearson’s correlation coefficients between selected variables

	Age	BMI	ISS	Days to operation	Open fracture	Sacral fracture	Methods of reduction	Patient’s position
Distance reduced in axial view (postoperative)	0.02	0.128	0.138	0.007	0.136	− 0.289**	0.349**	0.151
Distance reduced in coronal view (postoperative)	− 0.213	0.071	0.184	0.2	0.116	− 0.440**	0.299**	0.231
Reduction quality (Matta)	− 0.114	0.178	0.151	0.252	0.239	− 0.08	0.165	0.159
Reduction quality (Lefaivre)	0.141	− 0.089	− 0.019	0.103	− 0.059	0.197	0.095	0.286

**Statistical significance

Table 2 shows the characteristics of the patients in the closed reduction group (C group) and open reduction group (O group). There were no differences in the chosen parameters between the two groups. Moreover, there was no difference in the occurrence of sacral fracture between the two groups.

**Table 2:** Demographic data of patients who underwent percutaneous fixation using an open or a closed approach

	C group	O group	*p*
Number	46	10	
Age	40.61 ± 17.74	34.1 ± 15.42	0.71
BMI	25.23 ± 4.52	23.61 ± 5.08	0.54
Days to operation	8.33 ± 6.22	7.9 ± 2.69	0.21
Injury severity score (Median)	20 (IQR 13–27)	18 (IQR 8–20)	0.44
New injury severity score (Median)	23 (IQR 17–29)	20 (IQR 8–27)	0.47
Sex			0.65
Male	32	7	
Female	14	3	
Mechanism			
Motorbike accident	26	2	
Car accident	2	3	
Fall	5	2	
Crush injury	6	1	
Pedestrian-related accident	7	1	
Other	0	1	
Open fracture			0.71
No	41	9	
Yes	5	1	
Sacral fracture			0.19
No	27	8	
Yes	19	2	

*IQR* Interquartile range, *BMI* Body mass index

**p < 0.05 indicates statistical significance

Perioperative comparisons between the two groups are shown in Table 3. More patients were classified as AO B2.3 in the C group (*p* = 0.002). A significantly higher rate of prone position for surgery was observed in the O group (*p* < 0.001). More blood loss and a longer operation duration were observed in the O group than in the C group. Although real-time fluoroscopic guidance is essential for percutaneous ISS or TITSS, especially for the C group, we did not find a significant difference between the two reduction techniques. Regarding the measured ADIO of the screws, there were no differences between the two groups despite a larger angle measured in the O group.

**Table 3:** Perioperative diameters

	C Group	O Group	*p* value
Classification			0.002**
61-B2.3	33	2	
61-B3.1	0	1	
61-B3.3	2	3	
61-C1.2	8	4	
61-C2.1	1	0	
61-C2.2	1	0	
61-C2.3	1	0	
Operation position			< 0.001**
Supine	44	2	
Prone	2	8	
Estimated blood loss (mL)^#^	207	828	< 0.001**
Operating time (h)^†^	2.9	5.5	0.008**
Radiation exposure (mGy)	45.67 ± 14.42	38.45 ± 19.35	0.07
ADIO on mpCT (degree)			
ISS			
Axial	9.55 ± 6.6	9.63 ± 5.88	0.71
Coronal	5.57 ± 4.37	3.99 ± 2.58	0.17
TITS			
Axial	2.62 ± 2.54	3.48 ± 4.31	0.25
Coronal	1.71 ± 1.18	0.98 ± 0.56	0.18

*ADIO* Angles deviated from the ideal orientation, *mpCT* Multiplanar computed tomography, *ISS* Iliosacral screw, *TITSS* Trans-iliac-trans-sacral screw

^#^Blood losses with the anterior and posterior approaches were included in the estimation

^†^The operating time is the sum of the times required for (1) preparatory tasks carried out by the anesthesiology team, (2) patient positioning on the surgical table, (3) setup of intraoperative fluoroscopy, (4) execution of the EUA procedure, and (5) wound coverage

**p < 0.05 indicates statistical significance

The results of the postoperative imaging assessments are shown in Table 4. Both Matta and Lefaivre criteria revealed equal results for the two applied approaches. Upon mpCT assessment, wider SIJ distances before and after osteosynthesis were observed in the O group. However, SIJ distances were also significantly reduced in the O group in both the axial and coronal planes (*p* = 0.008 and 0.025, respectively).

**Table 4:** Radiological outcomes

	Closed	Open	*p*
Radiographic assessment			
Matta criteria			0.25
Excellent	27 (59%)	3 (30%)	
Good	14 (30%)	5 (50%)	
Fair	5 (11%)	2 (20%)	
Poor	0	0	
Lefaivre criteria			0.87
Excellent	14 (30%)	3 (30%)	
Good	13 (28%)	3 (30%)	
Fair	14(30%)	2 (20%)	
Poor	5 (11%)	2 (20%)	
mpCT assessment			
SIJ distance (mm)			
Before osteosynthesis			
Axial	5.81	11.32	< 0.001**
Coronal	5.54	9.67	< 0.001**
After osteosynthesis			
Axial	3.6	6.3	< 0.001**
Coronal	3.37	5.37	0.001**
Distance reduced			
Axial	2.21	5.02	0.008**
Coronal	2.17	4.3	0.025**

*mpCT* Multiplanar computed tomography, *SIJ* Sacroiliac joint

**p < 0.05 indicates statistical significance

**Fig. 5:**
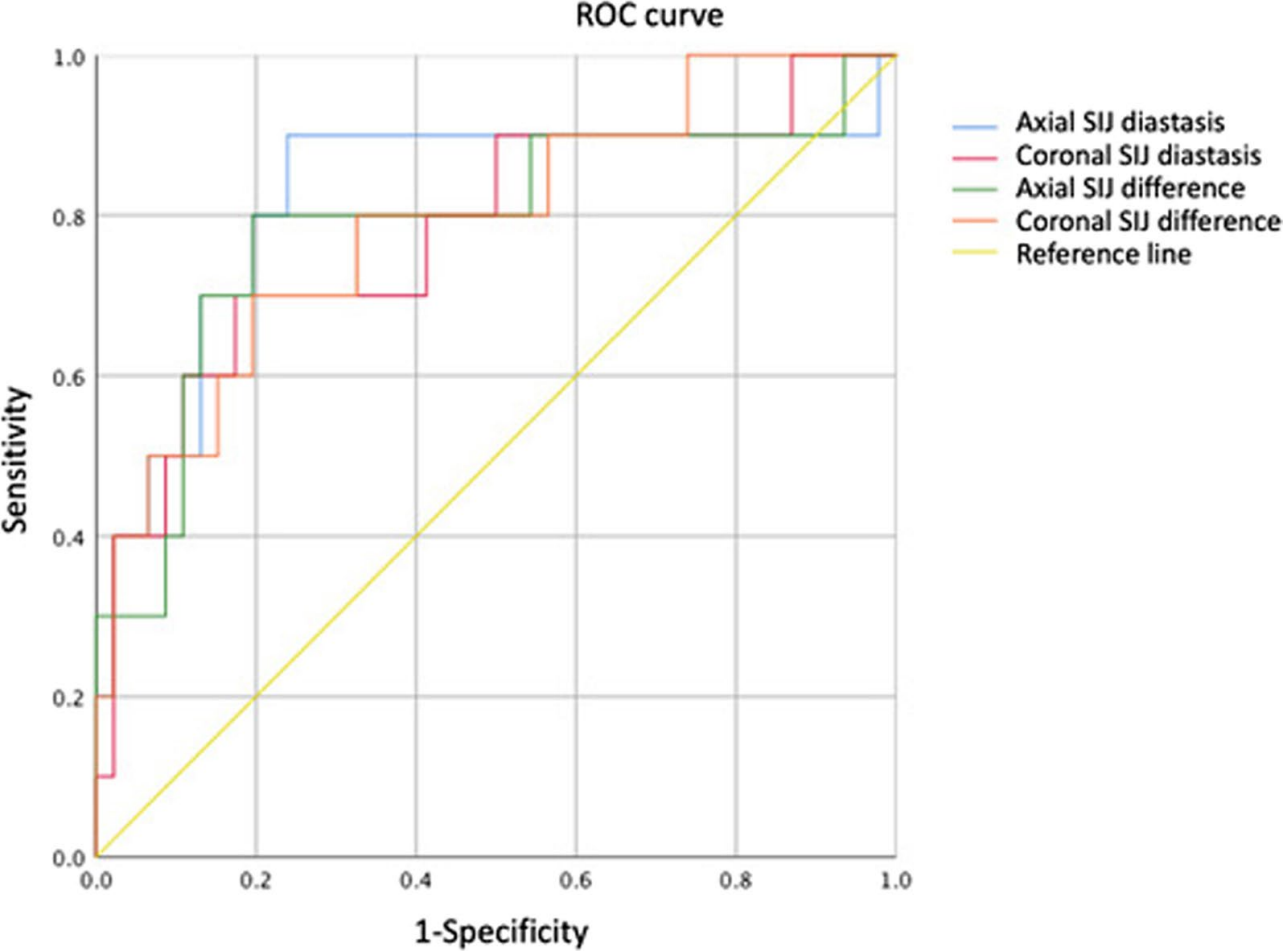
Receiver operating characteristic (ROC) curve

Both closed and open reduction could achieve good reduction quality, with more SIJ distance reduction observed in the O group (Tables 3, 4). We obtained a receiver operating characteristic (ROC) curve (Fig. 5) to determine if any listed factors, including preoperative SIJ diastasis in the axial/coronal view and preoperative axial/coronal SIJ joint difference between the healthy and injured sides, could predict whether the patients should undergo open or closed reduction. The four factors mentioned above showed an excellent or acceptable discrimination to predict open reduction with an area under the curve (AUC) of 0.822, 0.778, 0.789, and 0.791, respectively.

By calculating the maximum Youden’s index of the four factors mentioned above, we could identify the cut-point of SIJ diastasis that could predict patients who received open reduction and internal fixation (ORIF) (axial SIJ = 6.52 mm, coronal SIJ = 7.26 mm, axial SIJ difference = 3.39 mm, coronal SIJ difference = 3.71 mm). We assumed that if the patients, whose injured SIJ was wider than the cut-point distance, received closed reduction and internal fixation (CRIF), the distance reduced after osteosynthesis would be lesser than that in patients who received ORIF. According to our data, a preoperative SIJ difference of > 3.71 mm between the healthy and injured sides in the coronal plane showed increased SIJ reduction under open reduction with statistical significance (2.18 mm and 6.58 mm in closed and open reduction, respectively, with *p* = 0.021) (Table 5). The Matta and Lefaivre criteria were used to evaluate differences between CRIF and ORIF, with no statistical significance.

**Table 5:** Axial and coronal SIJ distance reduction using ORIF or CRIF

Before osteosynthesis	Reduced SIJ distance	
	Reduced axial SIJ distance (mm)	Reduced coronal SIJ distance (mm)
Axial SIJ > 6.52 mm		
CRIF	5.72	4.36
ORIF	5.46	4.29
Coronal SIJ > 7.26 mm		
CRIF	5.57	6.58
ORIF	6.04	4.94
Axial SIJ difference > 3.39 mm		
CRIF	5.58	4.02
ORIF	5.72	4.5
Coronal SIJ difference > 3.71 mm		
CRIF	2.18**	2.12
ORIF	6.58**	4.99

*SIJ* Sacroiliac joint, *ORIF* Open reduction and internal fixation, *CRIF* Closed reduction and internal fixation

**p < 0.05 indicates statistical significance

## Discussion

Satisfactory treatment of SIJ diastasis poses a great challenge for orthopedic surgeons. If SIJ diastasis is left untreated or undertreated, global pelvic instability may occur and cause short-term and possibly further long-term sequelae. CRIF with ISS, with or without TITSS, is an attractive treatment for diastatic joints because it is less invasive. However, ORIF may be needed when there is an imperfect reduction in the SIJ during screw purchasing. There is no evidence to explore the absolute indication for ORIF in cases of SIJ diastasis. We analyzed 56 patients who underwent percutaneous screw fixation with closed or open reduction. With our results showing a satisfactory screw position and acceptable reduction quality of the PRI, we found a critical difference in SIJ distance between the injured and healthy side (3.71 mm, coronal plane of the mpCT), which is recommended for open reduction of the injury.

Among patients with SIJ injury indicated for surgery, those who were surgically treated showed a better outcome and health-related quality of life [28–32]. To achieve anatomical reduction, different reduction methods and implant choices have been advocated [33]. The anterior approach with plating has the advantage of providing direct visualization to the anterior SIJ and simultaneous access to symphyseal disruption [10, 34, 35]. However, large tissue dissection, especially the elevation of the iliacus from the ilium, may cause postoperative morbidities [25]. Meanwhile, iatrogenic lumbar nerve root injuries may be encountered during the plating procedure [36]. Similarly, a prominent implant and increasing surgical site infection may be followed by posterior plating in terms of tension band plate for the posterior pelvic ring [37].

Iliosacral screw was first introduced by Matta et al. for posterior pelvic ring fixation [25]. Despite some reports raising concerns that ISS may be associated with more intraoperative exposure to X-rays and neurologic damage [38], it became a popular fixation method because it is less invasive and results in less blood loss and a lower infection rate along with good stabilization [39–41]. Khaled et al. reported that over 90% of patients with posterior pelvic ring instability could achieve excellent or good radiology outcome after ISS treatment [42]. A closed reduction maneuver is suggested for ISS insertion to preserve blood supply to the bone, limit scarring in the surrounding soft tissues, cause less blood loss, and reduce morbidity from open reduction [21, 41, 43].

However, drawbacks and limitations exist with application of the closed reduction method for SIJ injury [44]. Katharina et al. reported failed anatomical reduction by closed reduction in 5 of 31 patients, resulting in poorer functional outcome [45]. In a study by Starr et al. [21], closed reduction failed in 2 of 27 patients with SIJ injury because of an extended duration between the time of injury and surgery as well as marked displacement. Consequently, open reduction through an anterior or a posterior approach plays a role in managing SIJ diastasis. Through the anterior approach, fragments and/or debris within the SIJ could be visualized, and the surrounding fibrous tissue could be easily removed [34]. Using the posterior approach, sacral fracture visualization and nerve root decompression could be performed simultaneously with SIJ anatomical reduction and fixation with a percutaneous IS screw [46, 47].

However, there is no clear indication for the use of open reduction over closed reduction to achieve widening of the SIJ diastasis. Lindsay et al. [5] presented a similar satisfactory reduction quality via both closed and open reduction for ISS insertion. Both methods can achieve excellent or good reduction quality; however, no definite indications were documented in the present study. Some studies suggested open reduction in case of failure of closed reduction [44, 45]. According to the results of this study, we proposed that with a distance difference wider than 3.71 mm between the injured and healthy SIJ in the coronal plane, the open method is recommended for SIJ reduction, either from the anterior or mostly from the posterior approach. To our knowledge, this is the first study to focus on the indication of open reduction for diastatic SIJ by screw fixation.

Despite efforts to avoid bias, this study has several limitations. First, it had a retrospective design. Second, the selection of closed or open reduction was primarily based on the surgeon’s experience. However, our results advise that inadequate reduction may be achieved by closed reduction if the SIJ diastasis exceeds the critical value. Third, comprehensive evaluation of the bone quality was not performed for each patient. One factor that led to a successful closed reduction was the linear compressive force exerted by the ISS. Closure of the SIJ may be imperfect if the sacrum density is weak. Finally, the functional outcomes of the enrolled patients have not been reported. The actual relationship between the reduction quality and post-surgery functional performance could not be explored. Further studies should be conducted using a prospective method, focusing on the selection of patients who have undergone open reduction based on an established algorithm. Furthermore, the functional outcomes should be reported, and the correlation with the algorithm should be examined.

## Conclusions

In conclusion, fixation of the SIJ with the ISS and TITSS, either through closed or open reduction, could attain satisfactory and comparable radiological outcomes. Furthermore, for patients in whom the distance between the injured and healthy SIJ is > 3.71 mm in the coronal plane, surgeons should be prepared to perform open reduction (including ensuring the availability of sterile draping and appropriate reduction instruments) in case of unsatisfactory reduction by the closed method.

## Data Availability

The datasets used and/or analyzed during the current study are available from the corresponding author on reasonable request.

## Abbreviations

SIJ: Sacroiliac joint
ISS: Iliosacral screw
TITSS: Trans-iliac-trans-sacral screw
PRI: Pelvic ring injury
AP: Anteroposterior
mpCT: Multiplanar computed tomography
AO: Arbeitsgemeinschaft für Osteosynthesefragen
ADIO: Angles deviated from the ideal orientation
ROC: Receiver operating characteristic
AUC: Area under the curve
ORIF: Open reduction and internal fixation
CRIF: Closed reduction and internal fixation
